# Genebank Operation in the Arena of Access and Benefit-Sharing Policies

**DOI:** 10.3389/fpls.2019.01712

**Published:** 2020-01-22

**Authors:** Martin Brink, Theo van Hintum

**Affiliations:** Centre for Genetic Resources, the Netherlands (CGN), Wageningen University & Research, Wageningen, Netherlands

**Keywords:** genetic diversity, conservation, genebanks, Access and Benefit-Sharing (ABS), Convention on Biological Diversity (CBD), International Treaty on Plant Genetic Resources for Food and Agriculture (ITPGRFA), Nagoya Protocol

## Abstract

Since the 1990s, the exchange of genetic resources has been increasingly regulated. The Convention on Biological Diversity (CBD), the International Treaty on Plant Genetic Resources for Food and Agriculture (ITPGRFA) and the Nagoya Protocol recognize that countries have sovereign rights over their genetic resources and provide a framework for domestic legislations on Access and Benefit-Sharing (ABS). However, within the rules of these international agreements, countries can follow their own interpretations and establish their own rules and regulations, resulting in restricted access to genetic resources and limited benefit-sharing, effects that are contrary to the objectives of these agreements. Although the ITPGRFA’s Multilateral System of Access and Benefit-Sharing provides opportunities for easier access to plant genetic resources for food and agriculture (PGRFA), plant genebanks face increasing complexity in their operation. Adding material to genebank collections has become more difficult, not only because collecting missions need to be negotiated with national and local authorities, but also because acquiring material from other collections is only possible if the origin of the material is properly documented and is done in compliance with regulations. Genebanks may only provide access to their own collections if the material that is to be released is distributed in compliance with a) the conditions under which the material was received and b) the national laws of the country where the genebank is located. The only way genebanks can deal with this new complexity, apart from ceasing to add or distribute material, is by setting up proper procedures to document the origin of every accession and the conditions for their use and further distribution. To prevent a further decrease in access to PGRFA, complexity must be fought. Applying the ITPGRFA’s Standard Material Transfer Agreement (SMTA) only, even for material that does not fall under the ITPGRFA, would simplify matters. The scope of the ITPGRFA could be expanded to include all crops. Furthermore, certain ambiguities (e.g. regarding *in situ* material and wild species) could be resolved. Finally, compliance with the ITPGRFA should be improved and better monitored.

## Introduction

Plant genetic resources (PGR) include cultivated varieties, obsolete varieties, landraces, wild species (including crop wild relatives), breeders’ lines, research populations and mutants. Only a small proportion of all the available PGR are used, and humans depend on a very limited number of crops, in particular wheat, rice and maize, for the largest part of their caloric intake (McCouch et al, 2013; Khoury et al., 2014). The development and expanding cultivation of modern crop cultivars has led to decreased genetic diversity within crops (Langridge et al, 2006; Feuillet et al, 2008; Rufo et al., 2019). This loss of genetic diversity could make adaptation of crops to changing environmental conditions more difficult. Increased temperatures and changing rainfall patterns will cause geographic shifts in suitable cropping areas, and currently well-adapted crops or cultivars may become less adapted or even unsuitable for cultivation. Diversity is needed for crossing and selection, and diversity between and within crops (e.g. by using landraces and crop wild relatives in crop breeding programs) will need to be exploited in order to respond to climate change and to meet future food security challenges (Jump et al., 2009; Ramirez-Villegas et al., 2013; Lopes et al., 2015; Dempewolf et al., 2017; Zhang et al., 2017).

With regard to the conservation of PGR diversity, *in situ* and *ex situ* conservation can be distinguished. *Ex situ* conservation is defined as: “the conservation of components of biological diversity outside their natural habitats”, and *in situ* conservation as: “the conservation of ecosystems and natural habitats and the maintenance and recovery of viable populations of species in their natural surroundings and, in the case of domesticated or cultivated species, in the surroundings where they have developed their distinctive properties” (UNEP, 1992). In *ex situ* conservation, the diversity in stored material is fixed because the material is not subject to further natural selection or selection by farmers. (Obviously, while every regeneration of the material will result in slight changes in the genetic composition, maintaining the ‘genetic integrity’ of the accessions is the goal of *ex situ* conservation.) *In situ* conservation, on the other hand, is more dynamic, but is threatened by climate change and the resulting genetic erosion that can be expected to occur (e.g. Peñuelas et al, 2018). Therefore, the two types of conservation are viewed as complementary.

Genebanks conserve PGR under *ex situ* conditions, make them available for current use and keep them available for future use. As PGR diversity is the foundation of food security and climate resilience, genebanks play an important role in addressing the effects of climate change and other challenges to food security (Pellegrini and Balatti, 2016; Fu, 2017; Westengen et al., 2018). While genebanks previously catered above all to the demands of plant breeders, they have become more involved in long-term conservation and the distribution of PGR material to a wider range of users (Westengen et al., 2018). Some genebanks, such as the Centre for Genetic Resources, the Netherlands (CGN), have evolved into genetic resource centers. They carry out not only *ex situ* conservation but also *in situ* conservation as well as providing services to support PGR users in finding, selecting, obtaining and using PGR.

Users of PGR from genebanks often look for specific traits, such as drought tolerance, resistance to diseases or pests, yield potential, or levels of nutrients or other compounds, e.g. for use in a breeding program. They may also seek diversity for one or more traits for use in a scientific study of a particular trait. Genebanks or plant genetic resources centers help the user to identify the most suitable material and obtain it. Because of ease of access, the primary source of this material often is the collection of the national genebank, followed by other genebanks or *ex situ* sources, and finally *in situ* sources, including natural habitats for crop wild relatives and land of farmers or hobby growers for cultivated material.

However, since the 1990s, obtaining PGR for inclusion in genebanks and further distribution to breeders and other users has become increasingly difficult. Awareness of the actual or potential value of PGR has grown, and as a result an increasing number of countries are asserting their rights to genetic resources. The concept of Access and Benefit Sharing (ABS) was introduced, with ABS being defined as the regulation of access to and utilization of genetic resources and the sharing of the benefits arising from this utilization among users and providers. International ABS agreements were negotiated establishing that states can exercise rights over their genetic resources. This awareness and the resulting agreements have translated into well-structured regulation of access to PGR through domestic legislation in a number of countries. In other countries, however, it has resulted in confusion regarding access to PGR pending the legislative process, or confusion because of the complexity of the regulations.

This article describes the main international ABS agreements concerning PGR (*International Access and Benefit-Sharing Agreements Relevant for PGR*), the implications of these agreements (*Implications*) and the ways genebanks cope with these implications (*How Genebanks Cope*). In the final section (*Recommendations and Conclusions*) the authors outline some recommendations and conclusions.

## International Access and Benefit-Sharing Agreements Relevant for PGR

### The Convention on Biological Diversity (CBD)

The three objectives of the Convention on Biological Diversity (CBD, www.cbd.int/convention/), which came into force on 29 December 1993, are the conservation of biological diversity, the sustainable use of its components, and the fair and equitable sharing of the benefits arising from the utilization of genetic resources (UNEP, 1992). In the text of the CBD, genetic resources are defined as: “genetic material of actual or potential value”, while genetic material is defined as: “any material of plant, animal, microbial or other origin containing functional units of heredity” (UNEP, 1992).

Prior to the CBD, PGR were generally seen as a common heritage of mankind; PGR were usually freely collected, used, and transferred to other countries. The CBD, however, established that states can exercise control over the genetic resources in their territories. According to the CBD, prior informed consent of the party providing the resources is needed for access to genetic resources (unless that party has decided otherwise) and use and benefit-sharing must be done according to mutually agreed terms.

Although the CBD is primarily focused on wild biodiversity, it also affects the exchange of plant genetic resources for food and agriculture (PGRFA). The special role of PGRFA was recognized at the Conference for the Adoption of the Agreed Text of the CBD, held in Nairobi in 1992, when a resolution was adopted stating that solutions were to be sought for matters concerning PGR. This would in due time result in the establishment of the International Treaty on Plant Genetic Resources for Food and Agriculture (ITPGRFA).

### The International Treaty on Plant Genetic Resources for Food and Agriculture (ITPGRFA)

To address PGRFA in the post-CBD era, the FAO drafted and adopted the International Treaty on Plant Genetic Resources for Food and Agriculture (ITPGRFA, www.fao.org/plant-treaty), which came into force on 29 June 2004 (FAO, 2002). The objectives of the ITPGRFA are very similar to those of the CBD but focus on PGRFA: the conservation and sustainable use of PGRFA and the sharing of the benefits arising from their use (FAO, 2002). PGRFA are defined as: “any genetic material of plant origin of actual or potential value for food and agriculture” (FAO, 2002). The ITPGRFA confirms the sovereign rights of countries over their genetic resources but aims to facilitate the exchange of PGRFA by the establishment of a Multilateral System of Access and Benefit-Sharing (MLS) in which PGRFA are exchanged under a Standard Material Transfer Agreement (SMTA), instead of under the prior informed consent and mutually agreed terms prescribed by the CBD.

The MLS is a global pool of PGRFA, meant to facilitate access to these PGRFA as well as to achieve fair and equitable sharing of the benefits arising from their utilization. PGRFA may be added to this pool by countries and the institutions under their control, by natural and legal persons in the contracting parties and by international institutes (Manzella, 2013). The MLS does not extend to all PGRFA but covers a set of 35 food crops and 29 forages, which are listed in Annex I of the ITPGRFA. The selection of this set of crops and forages was based on criteria of food security and interdependence and was a negotiated compromise between countries favoring the inclusion of all PGRFA and countries favoring the inclusion of only a limited number of crops (Visser, 2013). According to Article 11 of the ITPGRFA, the MLS is to include all PGRFA of the food crops and forages listed in Annex I that are “under the management and control of the Contracting Parties and in the public domain” (FAO, 2002). PGRFA that belong to the food crops and forages listed in Annex I but do not fulfil the other conditions are not automatically included in the MLS but can be included on a voluntary basis by natural and legal persons holding these PGRFA. Access to materials in the MLS under the SMTA is granted only for their use in research, breeding and training for food and agriculture; other uses are explicitly excluded (FAO, 2002).

With regard to benefit sharing, the Contracting Parties to the ITPGRFA recognize that facilitated access itself is an important benefit, but also underline the importance of other forms of benefit sharing, such as the exchange of information, technology transfer, capacity building, and the sharing of commercial benefits. If material received under an SMTA is used to create PGRFA that are not freely available for research and breeding by others, the recipients must pay 0.77% of the sales of those PGRFA (or 0.5% of all sales of PGRFA belonging to the same crop) to an international benefit-sharing fund (www.fao.org/plant-treaty/areas-of-work/benefit-sharing-fund), which is used to support conservation and sustainable utilization of PGRFA. While information on the projects funded is available on the website, other information, e.g. on financial contributions, is missing.

The Contracting Parties to the ITPGRFA undertake to include in the MLS those PGR of the crops and forages in Annex I that are in the public domain and under their management. However, even if material is not part of the MLS, providers of PGR can distribute their material under the SMTA. The CGIAR centers make more than 750,000 accessions available under the MLS (FAO, 2019).

### The Nagoya Protocol

The “Nagoya Protocol on Access to Genetic Resources and the Fair and Equitable Sharing of Benefits Arising from their Utilization to the Convention on Biological Diversity” (UNEP, 2011, www.cbd.int/abs) entered into force on 12 October 2014. The Nagoya Protocol is a supplement to the CBD and is intended to improve the implementation of the benefit-sharing provisions of the CBD. Its objective is similar to the third objective of the CBD: the fair and equitable sharing of the benefits arising from the utilization of genetic resources. An Annex gives a long list of possible benefits (monetary and non-monetary) that can be shared.

The Nagoya Protocol not only applies to genetic resources as defined by the CBD, but also contains provisions regarding traditional knowledge associated with genetic resources. It defines the utilization of genetic resources as: “research and development on the genetic and/or biochemical composition of genetic resources, including through the application of biotechnology as defined in Article 2 of the Convention”. A major element of the Protocol is that, when genetic resources are used in their territories, Parties must monitor compliance with the domestic ABS rules of provider countries. In addition, Parties to the Protocol must provide rules and procedures for clear and fair access. Each Party must designate a national focal point, responsible for making information available, and a competent national authority, responsible for granting access. An Access and Benefit-Sharing Clearing-House (https://absch.cbd.int/) was established as a means of sharing information related to ABS, including contact details of national focal points and competent national authorities, legislative, administrative and policy measures, and issued permits.

With regard to the relationship between the Protocol and other international agreements, Article 4 states that the Nagoya Protocol is not applicable to genetic resources which are covered by another, specialized international ABS instrument. However, as of November 2019, discussions on the criteria for identifying specialized international ABS instruments and on processes for their recognition had not been concluded, and no specialized instruments have yet been recognized officially under the framework of the Nagoya Protocol. In practice, the ITPGRFA is considered by many countries to be such an instrument, implying that for these countries material exchanges covered by the ITPGRFA are not subject to the rules of the Nagoya Protocol.

More recently, discussion has arisen as to whether digital sequence information (DSI) related to genetic resources should also fall under the international ABS agreements. In general, there is consensus that access to and use of DSI is extremely important for conservation and sustainable development. However, views diverge on whether access to DSI and the sharing of benefits from its use are currently fair and equitable. Countries have different opinions on whether and how access to DSI and benefit-sharing from its utilization should be regulated, and discussions are taking place under the framework of the international agreements. In the meantime, some countries have included DSI in their domestic PGR access legislation.

## Implications

After the CBD had come into force (1993), domestic ABS legislation was established in various countries, including the Philippines (1995), Costa Rica (1998) and Brazil (2001). Bilateral agreements between providers and recipients of PGR became the rule for gaining access to them (Carrizosa et al., 2004). However, each country was allowed to have its own interpretations and make its own procedures, which resulted in a complex situation, also due to the uncertainty on how to make access procedures, the costs, and the sometimes insufficient capacity of countries to do this properly. This complex situation sometimes discouraged potential users from seeking access to genetic resources. So, while domestic access and benefit-sharing policies were intended to support, rather than hinder, the sharing of PGRFA (Wynberg et al., 2012), this was often not the case. Adverse effects of CBD-based domestic ABS regulations on biodiversity research and international collaboration have been reported by various authors (Jinnah and Jungcurt, 2009; Neumann et al., 2018; Prathapan et al., 2018).

With regard to access to PGR, the ITPGRFA has been more effective than the CBD, even though not all PGR are incorporated in the MLS, and not all PGR in the MLS are easily available. As of mid-July 2019, more than 5.4 million samples had been distributed (of which 5.2 million from Annex I crops) under about 75,000 SMTAs (FAO, 2019). However, most of the MLS transfers (92%) concern distribution from the collections of the CGIAR centers. Many Contracting Parties to the ITPGRFA have not publicly confirmed which PGR materials in their countries are in the MLS, making it hard for potential users to know which PGR are available (Halewood et al., 2013a). Also, there is much ambiguity about the status of PGR not included in *ex situ* collections but occurring under *in situ* conditions. Bjørnstad et al. (2013) tested the extent to which facilitated access was functioning in practice by sending requests for seeds to 121 countries which were Contracting Parties to the ITPGRFA. They received seeds from 44 of these countries, with 54 countries not responding and contacts with the other 23 countries not resulting in obtaining the seeds requested.

Concerning benefit-sharing under the framework of the ITPGRFA, significant non-monetary benefits have been shared through exchanged material, collaborative research, capacity building, information exchange, and knowledge creation. However, the MLS has hardly been able to generate any monetary benefits based on the use of the SMTA. This may be due to the considerable time that elapses between access to PGRFA and the commercialization of products based on these PGRFA. Also, the SMTA only makes benefit-sharing payments mandatory when the further use of improved material (based on material from the MLS) for research and breeding purposes is restricted (usually through patents). In practice, however, users of material from the MLS do not generally restrict access for research and breeding, and therefore are not subject to mandatory sharing of monetary benefits. A third factor is that some important crops, such as coffee, soya bean, sugarcane and tomato are not included in the current MLS. Much research is done on theses crops, which could have generated mandatory benefit-sharing. Consequently, the benefit-sharing fund of the ITPGRFA has mainly been filled by voluntary donor country contributions. The first mandatory payment to the benefit-sharing fund of the ITPGRFA was only made in 2018, when a Dutch plant breeding company (Nunhems, at the time owned by Bayer) paid about USD 120,000 (0.77% of the US sales revenues of seeds of ten vegetable cultivars developed using material obtained under the SMTA from genebanks in Germany and the Netherlands) (www.fao.org/plant-treaty/news/news-detail/en/c/1143273/).

Discussions are presently being held among the Contracting Parties of the ITPGRFA on proposals to create a subscription system for the MLS to assure earlier and more monetary benefit-sharing. In addition, the possibility of extending ITPGRFA’s coverage from the food crops and forages currently mentioned in Annex I to include all PGRFA is being discussed.

Because the Nagoya Protocol has only relatively recently entered into force, it is too early to assess its implications for access to and utilization of genetic resources. However, widespread fear exists among users of genetic resources that the Protocol will have negative consequences, which are likely to include high transaction and administrative costs, reduced access to genetic resources, reduced international collaboration and negative impacts on scientific research and public health (Watanabe, 2015; Comizzoli and Holt, 2016; Cressey, 2017; Deplazes-Zemp et al., 2018; Neumann et al., 2018; Ribeiro et al., 2018). Although all countries have national sovereignty over genetic resources, some Parties to the Protocol have opted not to exercise this national sovereignty and not to require prior informed consent and mutually agreed terms for access to their genetic resources. Other countries, while having developed access legislation, have given PGRFA a special status, with facilitated access.

When genetic resources are utilized in Parties to the Nagoya Protocol, these Parties are obliged under the Protocol to monitor compliance with ABS rules in the provider countries of these genetic resources. In the EU countries, for instance, this obligation is implemented though EU Regulation 511/2014 (the EU ABS Regulation) (European Commission, 2014). The EU ABS Regulation applies to genetic resources accessed on or after 12 October 2014 in a country that at the time of access was a Party to the Nagoya Protocol and had established access measures. To fall under the EU ABS Regulation, these genetic resources must be used in the EU in basic research, applied research and/or development on their genetic and/or biochemical composition (European Commission, 2016). Where the use of genetic resources falls under the EU ABS Regulation, users must perform due diligence to ensure that the genetic resources they utilize were acquired in compliance with the domestic ABS legislation of the provider country.

Although no specialized international ABS instruments have been recognized yet under the overall framework of the Nagoya Protocol, the ITPGRFA has been explicitly recognized as such in the EU ABS Regulation. This means that PGRFA included in the MLS and acquired from Parties to the ITPGRFA do not fall under the EU ABS Regulation. PGRFA transferred under an SMTA from CGIAR centers do not fall under the EU ABS Regulation either. If non-Annex I PGRFA were obtained under an SMTA from a Party to the Nagoya Protocol that has officially declared that non-Annex I PGRFA under its control can also be transferred under an SMTA, the user of these PGRFA has fulfilled the due diligence obligations of the EU ABS Regulation.

## How Genebanks Cope

Genebanks acquire most new PGR either through collecting missions or through answered requests from other collections. Both channels have become more difficult due to increased regulation. Collecting material from *in situ* sources has become very difficult in many countries. Collecting missions need to be negotiated with national and local authorities, but the procedures and responsibilities within provider countries are often unclear and efforts to gain more clarity are often unsuccessful because those responsible do not respond or are not prepared to make decisions. Obtaining material from other collections is only possible if the material is made available from these and if the origin of the material is properly documented and complies with ABS rules.

With regard to the distribution of material from genebanks, access to their collections can only be provided when distribution complies with the conditions under which the material was received and the domestic legislation of the country where the genebank is located. For instance, if a genebank acquires material under the condition that it can only be used for non-commercial purposes, the genebank cannot make this material available for commercial breeding.

Genebanks are therefore faced with more and more complexity in their operation. The only way genebanks can deal with this, apart from stopping acquisition or distribution of material, is by setting up procedures to properly document the origin of every accession and the conditions for its use and further distribution. This information needs to be made available to potential users. Genebanks will also need to use and store Material Transfer Agreements (MTAs) when material is distributed from the genebank to users. As a result, the volume of paperwork required in the material distribution process has increased significantly.

Apart from the increased complexity of genebank management and the associated costs, the decreased access to PGR is also affecting collaboration between genebanks. Genebanks will be less eager to rationalize their own collections by reducing duplication with other genebanks since they cannot be sure of access to other collections in the future. Indeed, countries might see a need to stockpile PGR material to ensure future access to it for their own research organizations and breeders, resulting in redundancies and a further stress on the already limited capacity of the PGR community.

On the positive side, as the large majority of the users of PGR from genebanks are involved in research, breeding and training for food and agriculture, PGR genebanks often can make use of the MLS of the ITPGRFA, which provides opportunities for facilitated access to PGRFA. This facilitated access involves the use of a standardized contract (SMTA) and procedures, instead of the bilateral, case-by-case contracts and procedures arising from the CBD and Nagoya Protocol. Furthermore, the SMTA can also be used for non-MLS material. The European Cooperative Programme for Plant Genetic Resources (ECPGR) stated in 2016 that “It is recommended that all ECPGR member countries, as appropriate and in line with national legislation, use the SMTA for distribution of both Annex I and non-Annex I PGRFA accessions independently of whether material is conserved in *ex situ* collections or held *in situ*.” (ECPGR, 2016). Various countries have already declared that PGR under their management and control and in the public domain are made available by them under the SMTA, irrespective of whether these PGR are of a species contained in Annex I of the ITPGRFA.

In the EU, the ABS Regulation that implements the compliance aspects of the Nagoya Protocol applies to the utilization of genetic resources and not to their possession. The Guidance published by the EU explicitly states that activities such as the management of a collection for conservation purposes are not considered to be utilization. However, genebanks typically aim at making genetic resources available for utilization in breeding and other research and development activities. Therefore, it is good practice for genebanks to support users through seeking, keeping and transferring all relevant information, including access permits and contracts. Furthermore, for genebanks to operate legally, acquisition of PGR should be done in line with the access requirements of the provider country.

The Centre for Genetic Resources, the Netherlands (CGN), a genetic resource center managing the Dutch national plant genebank, may serve as a specific example of how genebanks cope with increased regulation of access to PGR. CGN operates on two principles: follow the rules and be transparent. The aim is that the origin of all PGR in the CGN collection and the legal basis of their acquisition are traceable. CGN distinguishes three categories of PGR germplasm in relation to its legal status (www.wur.nl/en/show/Access-and-beneft-sharing-Status-of-CGN-collections.htm):

1. Genetic resources of crops listed in Annex I of the ITPGRFA and forming part of the MLS. Access to these collections is provided under the SMTA of the ITPGRFA;
2. Genetic resources not listed in Annex I of the ITPGRFA and acquired by CGN before the CBD entered into force. In principle, CGN will provide this germplasm to the user under the SMTA, unless contractual obligations agreed upon during acquisition of the material by CGN require additional conditions;
3. Genetic resources not listed in Annex I of the ITPGRFA and acquired by CGN after the CBD entered into force. These are subject to the national sovereignty of the country of origin. Where possible, CGN provides access to these genetic resources under the SMTA, but where needed, CGN adapts the SMTA to incorporate additional conditions set by the provider country or contractual obligations agreed upon during acquisition of the material by CGN.

The “regular” CGN collection contains about 23,000 accessions of a range of agricultural and horticultural crops. In addition to its regular collection, CGN also offers seed samples from “special collections” that have been developed for a specific purpose targeting specific user groups, such as a collection of 73 re-sequenced tomato lines and a collection of 470 single seed descent (SSD) lines of *Lactuca* spp. CGN’s aim is to be able to make its regular PGR collection available in perpetuity, with all material being fully and freely available under SMTA (where it is to be used for research, breeding, or training for food and agriculture purposes), as the use of the SMTA reduces complexity and the free availability reduces transaction costs.

To achieve this, CGN makes all possible efforts to acquire and only include in its collection material that can be distributed in this way. This means that collecting missions are undertaken after signing an agreement in which the Competent National Authority of the country where the collecting takes place agrees with the subsequent distribution of collected material by CGN under the terms and conditions of the SMTA. The modalities are laid down in a Memorandum of Understanding between CGN and the Competent National Authority of the country of collection. The Memorandum of Understanding may cover a single collection mission or various collection missions over an extended period of time, as CGN strives to establish multiyear collaborations with countries. With respect to the benefit-sharing component of the agreements, CGN aims to include a substantial capacity development component. This may, for instance, include participation of representatives of the provider country in international courses on the conservation and use of PGR or the organization of tailor-made PGR courses in the provider country itself by CGN staff.

Unfortunately, not all countries or institutes from which CGN would like to acquire material are willing to allow incorporation of these PGR in the CGN genebank under the conditions of the SMTA. Some countries, for instance, are not comfortable with the associated multilateral character of monetary benefit-sharing and prefer bilateral sharing of monetary benefits instead. In these cases, the material cannot be acquired under the conditions of the SMTA and will thus not be included in the regular CGN collection. Since the material might still be valuable to some users, the possibility of creating a special collection for that material exists, but this is only done in exceptional cases. These special collections with material that can only be distributed under additional conditions are generally maintained on the principle of cost recovery, and access may need to be negotiated (possibly even with the donor of the material).

CGIAR genebanks too have been facing increasing difficulties in their efforts to acquire and conserve PGR in the past decades, for a large part due to ABS issues (Halewood et al., 2013b). The collections of the CGIAR genebanks have been placed in the MLS of the ITPGRFA, which means that the PGR included are available under the SMTA. As such, their activities are mainly governed by the ITPGRFA. However, when these genebanks want to acquire materials that are not included in the MLS, they have to comply with domestic access regulations based on the CBD and the Nagoya Protocol. Guidelines have been developed for the CGIAR genebanks on how to comply (CGIAR Genebank Platform, 2018).

## Recommendations and Conclusions

Policy developments since the 1990s that were aimed at regulating Access and Benefit-Sharing have so far resulted in reduced access. This is felt by genebanks, which face increasing difficulties in adding material to their collections, either through collecting missions or by obtaining material from other collections. As genebanks play a key role in conserving and making available key resources to address climate change and other challenges to food production and food security, this is an undesirable and possibly even dangerous development. Given climate change and the resulting genetic erosion that can be expected to occur, collecting and subsequent conservation in genebanks are essential for limiting losses of valuable PGR.

The national sovereignty of countries over their genetic resources has been firmly established and genebanks have to comply with the domestic access regulations in the countries where they collect material. As illustrated above, genebanks accept this and are fully committed to comply with domestic ABS measures. They are ready to share benefits, especially through capacity development in the countries where they collect. However, genebanks struggle with the complexity and unclarity of the way this sovereignty is exercised at the domestic and international levels.

To prevent a further decrease in access to PGRFA, this complexity must be fought. Although ABS issues are inherently complex, the resulting ABS regime should be kept as simple as possible. Acquisition and distribution of germplasm have to stay workable for genebanks as these activities play an important role in assuring the world’s food supply in the current times of climate crisis and population growth.

The ITPGRFA and its SMTA could play a key role in reducing complexity. The CBD and the Nagoya Protocol do not prescribe in detail how ABS should be implemented through domestic legislation and leave room for countries to decide for themselves how to exercise their sovereignty over their PGR. Applying the SMTA also to material not contained in Annex I to the ITPGRFA (and thus not in the MLS), would simplify matters. Various countries have already decided to do so for PGR under their management and control and in the public domain.

It should also be made clearer for potential users which PGR of Annex I crops and forages in ITPGRFA countries are available in the MLS. More clarity should be created on the status of *in situ* material in the context of the ITPGRFA. To achieve this, compliance with the ITPGRFA should be improved and better monitored. While the ITPGRFA has 145 member countries, only 54 national reports on the implementation of the ITPGRFA are available on the ITPGRFA website (www.fao.org/plant-treaty/areas-of-work/compliance/compliance-reports/en/). In comparison, for the Nagoya Protocol, which had 120 member countries as of 22 November 2019, 94 national implementation reports were available on the ABS Clearing house website on that date (absch.cbd.int/reports).

Contracting Parties to the ITPGRFA have been discussing expansion of the scope of the MLS of the ITPGRFA from the 64 food crops and forages mentioned in Annex I of the ITPGRFA to include all PGRFA. In addition, they have been considering the idea of creating a subscription system for the MLS to assure earlier and more monetary benefit-sharing. Expansion of the MLS would be a major development in the process of reducing the complexity. As the lack of sharing of monetary benefits from the utilization of materials provided through the MLS has remained an issue affecting the readiness of provider countries to allow access to their PGR through the MLS, the creation of a subscription system may not only result in more benefit-sharing but also in better access. Unfortunately, an agreement on the expansion of the MLS and the creation of a subscription system was not reached during the ITPGRFA Governing Board meeting held in November 2019. This was mainly due to diverging views as to whether access to DSI related to genetic resources from the MLS and benefit-sharing from its utilization should be regulated under the ITPGRFA.

In the end, it is in the interest of all countries that genebanks continue to be able to play their role of conserving and making available the key resources that are needed for meeting the demands of a growing world population in a changing climate.

## Author Contributions

MB and TH both contributed to the conception and design of the article. MB wrote the first draft of the manuscript, with TH writing additional sections. Both authors contributed to manuscript revision, read and approved the submitted version.

## Conflict of Interest

The authors declare that the research was conducted in the absence of any commercial or financial relationships that could be construed as a potential conflict of interest.
